# We all care just as much about the child: stakeholders’ experiences of parenting support in a Norwegian school context

**DOI:** 10.1080/17482631.2021.1908014

**Published:** 2021-04-08

**Authors:** Anita Berg, Lily Appoh, Kristin Berre Ørjasæter

**Affiliations:** Faculty of Nursing and Health Sciences, Nord University, Namsos, Norway

**Keywords:** Basic school, home-school collaboration, parenting support, qualitative study

## Abstract

**Purpose**: Despite increased focus on parenting support internationally, there is a lack of agreement in what constitutes parenting support. This paper explores the experiences of parenting support activities from the perspective of stakeholders in Norwegian basic schools.

**Methods**: Five focus group interviews were conducted with representatives from the schools’ parent work committee, class teachers, health nurses, and social workers from nine schools. The data were interpreted using an inductive thematic analysis.

**Results**: Three main themes emerged from the data: (1) A community for the best of the child, (2) uniting through relations, and (3) sharing knowledge and language. Parenting support was experienced as universal, relational, and multidimensional. It was related to everyday life interactions between the home, school, and the parenting community with the best interest of the child as a goal.

**Conclusion**: To avoid reducing parents to passive recipients of expert advice, parenting support activities should be an integral part of everyday school-home-parenting community collaborations.

**Abbreviation**: EU-The European Union; UN-The Unition Nations; CoE-The Council of Europe; NSD-The Norwegian Centre for Research Data; CTP-Class teachers in primary school; CTJS-Class teachers in junior secondary school; P-Parents; H-Health nurses; SW-Social workers

## Introduction

Bringing up children has been regarded as one of the most important tasks adults perform (Abela & Walker, 2013). Children learn the basics of social interaction from their parents and within their family units (Repetti et al., 2015). Over the years, research has emphasized the need to support parents in the effective performance of their parenting roles (Goodall, 2015).

On the international scene, several bodies, such as the European (EU) , the Council of Europe (CoE), and the United Nations (UN) are advocating for parenting support to be included in governmental policies (Daly, 2015). In 2018, the Norwegian government launched a four-year policy document captioned “Confident Parents—Confident Children: The Government’s Strategy for Parenting support (2018–2021)” (The Norwegian Directorate for Children Youth and Family Affairs, 2018). This policy emphasizes prevention as socio-economically profitable because it reduces the need for later and more costly measures. The government with this strategy aims to promote the child’s best interests by strengthening the relationship between parents and/or between parents and children, where parents should have and find support regardless of where they live.   

A distinction is commonly made between universal parenting support aimed at most parents, selective parenting support aimed at families that are at risk for one reason or another, and indicative parenting support aimed at families that already have an established challenge (Bråten & Sønsterudbråten, 2016). Traditionally the concept of parenting support has focused on the latter measure, which is helping parents in “need” and thereby putting them into roles as passive recipients of help from professionals to improve their childrearing abilities.   

Universal provision of parenting support has been claimed to be a unique feature of Nordic countries (Glavin & Schaffer, 2014). However, doubts have been raised concerning the accuracy of this claim. A review of the literature during the past decade has shown that most parenting support provided in Nordic countries, when measured by parental guidance programmes, are designed to meet the challenges of families at risk rather than being universal support (Appoh, 2019; Bråten & Sønsterudbråten, 2016; Rambøll, 2013; Sundsbø & Sihvonen, 2018). Studies of Wesseltoft-Rao et al. (2017) and Lundqvist (2015) suggest that parenting support in Nordic countries are more complicated and less top-down and mainstream than the policy suggests.

Despite the focus on parenting support internationally and nationally, there is a lack of agreement in what constitutes parenting support. According to Daly (2015), parenting support means different things depending on the context and the perspectives of practitioners, parents, and the rest of society. Watson et al. (2012) also indicated that understanding of parental support has practical consequences for children, parents, and professionals. The terminology used also varies, including “parent education”, “parental support”, “family/parent training”, and’ family/parent support’. For example, Hallberg (2006) provides a general definition of parental support as consisting of organized work with parents to promote the child’s well-being. A more detailed definition given by Daly and Bray (2015, p. 12) states, “Parenting support is a set of (service and other) activities oriented to improving how parents approach and execute their role as parents and to increasing parents’ childrearing resources (including information, knowledge, skills and social support) and competencies.”  

Parenting support may be provided to individuals or to communities through social networks (Sihvonen, 2020).  A social network can be seen as a web of social relations surrounding an individual and the characteristics of those ties (Berkman & Glass, 2000). Social support is a type of support given through social networks and seen as transactional which involves giving and receiving (Berkman & Glass, 2000; Richardson et al., 2007). Belsky (1984) suggests that social support in the context of parenting, functions in three general ways: By providing emotional support, for example, through caring actions; instrumental assistance, such as sharing information; and social expectations, which are guides on appropriate and inappropriate behaviour. Sihvonen (2018) underline that, within a community, parents’ own expertise and their peers’ horizontal expertise are as valuable as professional expertise when used as resources to support parents in their parental roles. 

Our review of the literature for this paper revealed that much of the available research is written from the perspective of the professionals who design and evaluate the programmes that are used in parenting support (Sundsbø, 2018) or from a policy-related perspective (Daly, 2015; Molinuevo, 2013; Littmarck et al, 2018). These are important and they have their place in the discourse on parenting support; however, there is little knowledge in the literature regarding how those engaged in parenting support activities in different contexts experience parenting support.

Children are seen as a part of various interdependent ecological systems, all of which affect the development of the child (Bronfenbrenner, 1989). Children spend most of their waking hours at school. The school and the home are presented as separate social institutions, yet firmly tied together for the purpose of upbringing children (Sihvonen, 2020).  According to Katz (2006), schools are in touch with more parents than any other institution in most societies and are thereby able to reach out to parents in a way no other agency can. The school is described as the natural home for parenting support as it probably is the only institution regularly engaging with the parents over the entire developmental phase of the children (Hodges & Healy, 2018). Together, schools and families can form caring communities (Epstein, 1995). There is a need for empirical studies exploring how the concept of parenting/parental support is interpreted and practiced in schools (Bergnéhr, 2015).

This paper explores the experiences of parenting support activities in a school context. Our aim is to contribute to the ongoing discourse on parenting support from the perspective of those engaged in the said parenting support activities. We do this this by presenting and discussing our qualitative findings that explored the experiences of parenting support by stakeholders in a basic school context in Norway.

## Methods

### Context

This study explores the experiences of parenting support in a Norwegian basic school context from the stakeholder’s perspectives. In Norway, children aged six to 16 are enrolled in basic school, which is divided into primary school (i.e., 1^st^–7^th^ grade) and junior secondary school (i.e., 8^th^–10^th^ grade). In this study, stakeholders refer to actors who have an interest in the affairs of the school, either by being a parent representative or staffs who, by virtue of their position, have active roles in the school-home collaboration.

This research forms part of a local intervention within the National Programme for Public Health Work in Municipalities (2017–2027). The data for this paper were generated on behalf of three Norwegian municipalities which merged to become officially one municipality in 2020. In their process of merging, the local project group wanted to create a common knowledge base to improve parenting support in their basic schools based on the experiences of those involved in parenting support activities. The new municipality has a population of 15 201 (Statistics Norway, 2020), nine school districts, 12 basic schools and a total of 1872 pupils. The included schools are either solely primary or junior secondary or combined primary and junior secondary schools.

### Recruitment and participants

To enter new research fields to collect data, key persons are of importance (Whyte, 1993). Gatekeepers are key persons with specific roles in a research situation where researchers need access to participants with specific characteristics (Wassenaar & Singh, 2016). In this study the local project group became our gatekeepers and our link to the stakeholders. Based on discussions within the local project group the public health coordinator and a project employee were asked to present the project in the monthly school leaders’ meeting and the municipal parent work committee meeting to identify and map the stakeholder groups who are involved in parenting support activities.

Based on this mapping the participants had to meet the following criteria: (i) They had to be appointed to the schools’ parent work committee or be employed as a health nurse, class teacher, or social worker; (ii) the employed staff should be involved in parenting support activities in their position for a minimum of two years and have had regular contact with parents of pupils in basic school. Such a selection strategy is described as purposeful sampling because only those who were directly involved in and were knowledgeable about the phenomenon of interest were invited to participate (Creswell & Plano Clark, 2011; Whyte, 1993). 

In recruiting participants from the stakeholder groups, the key persons used their local knowhow to combine the inclusion criteria with the desire of local project group to form a knowledge base representing all the school districts, types of schools and school sizes. Potential participants were requested to confirm their interest to participate in the study by phone followed by a written information sheet via email. Based on this ongoing recruitment process twenty-eight stakeholders agreed to participate and were included in the study. They included six participants from the schools’ parent work committee, five health nurses, seven class teachers in primary school, six class teachers in junior secondary school, and four social workers. The included schools had a number of pupils ranging from 44–268.

### Data collection

To address our research question of how parenting support was experienced among stakeholders in a basic school context, we used focus group interviews. Focus group interview is a well-known qualitative data collection method, which could be described as a form of group interview where a limited number of individuals are gathered to discuss a phenomenon (Savin-Baden & Major, 2013). In this study, five focus group interviews were conducted with homogenous groups (i.e., comprising parent representatives, social workers, health nurses, class teachers in primary school and class teachers in junior secondary school) to discuss stakeholders’ experiences regarding parenting support in a Norwegian school context. In stratified purposeful sampling (Patton, 1990), homogenous groups may enable different perspectives to emerge between groups if differences are found significant in the analysis.

The focus group interviews were carried out during the spring of 2019. Each focus group interview lasted between 90–120 minutes and included four to seven participants. A semi-structured interview guide was developed to prepare and conduct the focus group interviews (Kvale & Brinkmann, 2015). The interview guide consisted of loosely thematic open-ended questions covering topics such as participants’ understanding of parenting support, which type of support they were aware of and considered useful and supportive, and the understanding of their roles in relation to parenting support in basic schools. The interview guide was designed to give voice to participants and elicit responses concerning participants’ experiences and practices regarding parenting support. Some of the open-ended question asked during the interviews were as follows: How do you understand parenting support activities? Which parenting support activities are you aware of? How do you collaborate on parenting support activities? The focus group interviews were planned to be flexible and conversational. In order to establish a good dialogue without too many interruptions, the order of the questions was changed, if needed, during the interviews.

The focus group interviews were audio recorded and then transcribed verbatim by a professional transcriber. Each interview comprised an average of about 30 pages of text, giving a total of 148 pages of data.

### Data analysis

The inductive thematic analysis method by Clarke et al. (2015) was used to analyse the data. Our analysis was data-driven, which means that identified themes were closely linked to the data, known also as “bottom up analysis” (Clarke et al., 2015). The data analysis was carried out in six phases:

1. *Familiarization with the data*: The transcribed interviews were read several times to gain an overall understanding of the material in its entirety. All authors noted thoughts and reflections during their reading, shared their notes, discussed them, and reread the transcripts.
2. *Generating initial codes*: The interview text was broken into smaller parts. Workshops were conducted to discuss interesting quotes from each of the interviews. Further, initial codes were visualized in mind maps and tables using the Mindjet MindManager software (Corel Corporation, 2019). Through the mind maps, it was explored whether parenting support was experienced differently based on role. As this did not appear to be the case, further analysis was conducted on the whole dataset to bring out the overall experiences of parenting support.
3. *Searching for themes*: In this phase, the authors searched and identified themes by gathering all relevant data for each potential theme. Two main themes emerged at this stage: (1) practical support and (2) relational support.
4. *Reviewing themes*: During this phase, the authors in workshops discussed, reflected upon, compared, refined and merged the themes (see Table I for an example of the analysis process).
5. *Defining and naming themes*: Formulating themes was a process, and earlier themes were reconsidered in later stages of analysis. The authors held workshops where the themes were named and organized into main and subthemes. The following three main themes were defined and named: (1) a community for the best of the child, (2) uniting through relations and (3) sharing knowledge and language (see Table II).
6. *Producing the report*: Writing is an integral part of data analysis, not something that can only be done in the end (Clarke et al., 2015). During this phase, we have jointly written and re-written the text several times to present the findings.

**Table I.** An example of the data analysis process

Meaning unit	Condensation	Sub-theme	Main theme
Being taken seriously as a parent when you call the school, I think is very important; because, if you get rejected, then communication and parenting support are non-existent (P1)	To be taken seriously when contacting the school	Low threshold for contact	Uniting through relations

**Table II.** Overview of main and sub-themes

Main themes	Subthemes
A community for the best of the child	The best of the child a common goal Recognition that all parents need support Respect for children`s and families` differences
Uniting through relations	Low threshold for contact Common meeting places
Sharing knowledge and language	Knowledge of the developmental process of children Understanding childhood in contemporary society Access to local system knowhow A common language

In this paper, participants’ names were changed to numbers and letters to indicate whether they are parents (P), health nurses (HN), social workers (SW), and class teachers in primary school (CTP) or in junior secondary school (CTJS). To distinguish participants in each group, they have an identification number. Furthermore, before being translated into English, the quotes from participants were somewhat “language-washed” to increase readability.

### Ethical considerations

The Norwegian Centre for Research Data (NSD, 2019/987669) approved this study. Participation was voluntary and informed written consent was obtained prior to the focus groups interviews. Participants were informed that they could withdraw from the study at any stage. Data were processed in accordance with ethical guidelines provided by NSD and the current national guidelines and laws (Lovdata, 2017; National Commitee for Research Ethics in the Social Sciences and the Humanities, 2016).

## Results

In examining participants’ experiences of parenting support in a basic school context, our analyses revealed three main themes and eight sub-themes (see Table II). An elaboration of these themes follows.

### A community for the best of the child

Our study revealed that the experience of parenting support was described as a collaboration between the school, the home, and the parenting community. The child was the common focus, and there was the recognition that the contexts of the home, school, and leisure all have an impact on the child’s health, development, and well-being. Parenting support was viewed as a natural and necessary part of creating a community for the best of the child by the adults at home and at school. This community has three basic characteristics which correspond to the three subthemes: *The best of the child as a common goal; recognition that all parents need support; and respect for children’s and families’ differences.*

### The best of the child as a common goal

The best of the child was seen as fundamental to parenting support. Participants discussed and stressed during the interviews that the collaboration among the school, home, and parenting community is crucial for the development of the child. This can be seen in the quote by one of the class teachers in primary school when asked to define the goal of parenting support:

*Getting a good collaboration and work towards common goals for the benefit of the pupil* (CTP 2)

Parenting support was experienced as a reciprocal support and a collaboration by all the adults that are part of the multiple contexts that the individual child, and the children as a group, live in.

### Recognition that all parents need support

The need for support, albeit in different forms and degrees, was viewed as a natural need for all parents and not only for those in risk groups. Support is needed to build confidence in the parenting role and to cope with the challenges that may emerge during child rearing. The need for support was understood as a joint need for all parents, not just related to specific children, parents/families, or situations.

Participants underscored the importance of recognizing that all parents need support, even though it may seem like they have mastered the parenting role, as explained by one of the participants:

I think all parents need a little support from 1^st^ to 10^th^ grade (HN2)

Participants recounted that the authorities easily forget that parents need support throughout their parenting period, including when the children start primary school and, especially, when they become adolescents. In the view of the participants, as children reach new developmental phases, parents meet new challenges in their parenting roles:

*Some parents have asked why there is less parenting support when their children begin at school or come into adolescence. When the children were young, parents got close follow-up at the health station. When the children got older and the real challenges began, the parents experienced less support (…) Some need a lot of support and some need almost nothing. Those who seem to need nothing, I think they also feel good about growing in their parenting role* (HN 2)

Moreover, the type and degree of needed support differs; some need a short unsolicited confirmation of their parenting abilities through individual talks and guidance during everyday life challenges. Others need extensive follow-ups from school and other support agencies when experiencing challenging situations. All parents would like and benefit from being seen and confirmed as parents:

*In a way, they needed a little nod from us [the parents say things like] I’m not exaggerating, right? Is this real? They need to get some kind of confirmation (*CTJS 1)

Participants described that parenting support is about tailoring the follow-up with parents to the actual needs of individual parents, rather than being about the provision of the same type of support for all, independent of the individual’s situation. In other words, the support offered must meet the challenge being experienced.

#### Respect for children’s and families’ differences and needs

The experience of parenting support was also underpinned by a basic understanding that both children and their families are unique, live in different life contexts, and experience different challenges, at different times, and in varying degrees. These differences must be respected for parenting support to be appropriately experienced and exercised. Discussions about whether it was right to have common rules for the children made this visible:

Some schools create such common rules. I do not think that it is parenting support at all. We are individual families and we are single individuals. We must be free to choose what we want. (…) Respect one another; we are different (P1)

Although common rules could be helpful, they should only be viewed as guidelines in making the best decisions for the individual child and family. This respect was fundamental not just in the school-home interaction, but also in the parenting community:

*There was this issue about having a common rule for Snapchat. But, maybe, that is not the goal, but that parents should be strengthened in making their own choices?* (SW 3)

The purpose of parenting support was empowering the parents to make the best decisions for their children, rather than making similar decisions. Discussion of common rules could be helpful, but there should be no absolute rules that neglect the families’ individuality and autonomy.

### Uniting through relations

The second main theme shows that the established relationships of home-school and peer collaboration gives strength to all involved and consists of the two inter-related sub-themes: *low threshold for contact* and *common meeting places.*

#### Low threshold for contract

Low threshold for contact was found to be central in parenting support. Low threshold refers to the idea that parents should feel that they can contact the school, and the school can contact the parents, irrespective of how small or big the need for support might be. As one of the participants explained:

*Being taken seriously as a parent when you call the school, I think is very important; because, if you get rejected, then communication and parenting support are non-existent* (P 1)

Parents need to know whom to contact at the school, to have a relationship with these people, and they must be considered as available:

*Contact with the homes when the kids are struggling (…) just being seen and being heard. That someone calls the home to ask: Are you okay? That, in itself, kind of takes the burden off the parents, so they feel like they are almost halfway. Because they realise we know about them, we are engaged and aware of how the girl or boy is doing, and we are following up* (CTJS 3)

Knowing that there is someone at the school committed to their child and the family’s situation, who cares about their child and initiates contacts if necessary, and who is available when needed, was experienced as an important part of parenting support. This implies that adults at school and home who are around the child should be accessible to each other:

The parents may ask me, but I can also openly ask them if something is challenging for the pupil at school. They can give me advice or guidance, for example, on how to respond to the pupil in different situations, both difficult and positive ones. Likewise, I can give them tips on what I have tried at school that has worked out well, so that they may also try it at home. In that way, we can help each other (…) It is all about the same child, and we all care just as much about that child (CTJS 1)

However, to enable those involved around the child to work in the same direction, it was crucial that they had confidence in each other:

*But you have to be confident in each other, so that you can address things that may not always be positive. Then, there must be a low threshold for contact both ways, I think* (CTP 2)

Having a low threshold for contact was regarded as fundamental in parenting support by participants as it provides confidence to engage in dialogue when needed. Good relationships, in themselves, were seen as an investment that was preventative of conflicts and supportive of the child’s development. As participants highlighted, parents and staff at the schools must know each other and experience mutual trust. Then, they have the necessary basis for establishing relationships to work in the same direction for the best of the child.

#### Common meeting places

Participants highlighted the importance of having common meeting places. According to participants, some meeting places should be organized just for parents, some for parents and school staffs, and others for parents, school staffs, and children all together.

The confidence in the parenting role was associated with the existence of common meeting places, as they knowingly helped parents to get to know each other and to have an informal arena to discuss their parenting roles with peers. Being part of such a parenting community was experienced as important and empowering. Knowing that other parents, from time to time, also struggled with the same challenges when rearing their children gave parents confidence and acknowledgement that they were not alone:

Sometimes I wonder: Am I a bad mother now? (…) I think it is very important to have this social arena with other parents, so we can discuss. Because I don’t really think we are that different; we don’t disagree [in the goals of child rearing]. I think many of us struggle from time to time, so it’s okay not to be alone with these doubts (P 2)

Having access to a parenting community was perceived as a vital source of support. Participants expressed that having parents standing together as a parenting community works preventively. They had a belief that if children saw their parents talking together, it could prevent them from excluding or bullying other children. Then, parents, as a group, could act more easily and earlier when dealing with potentially problematic situations, without having to request professional intervention.

After all, I think it is important that we have an arena where parents can meet because I don’t think we always need a health nurse or milieu therapist to solve the problems for the kids. I think that when the kids see us meting and talking, we also avoid quite a lot of problems (P2)

Participants also emphasized the importance of having some formal meeting places for parents and school staffs. In Norway, it is common to have one or more parent meetings at each grade every year to inform about the school, the content of the education, how parents can get involved, routines, etc. However, participants reported that parenting meetings today are largely characterized by one-way communication, and emphasized the importance of establishing common meeting places that address the need for dialogue and reflection:

It may have to be on a different platform than parent meetings; arenas where one can actually have a two-way communication and reflection (HN1)

There was a consensus among participants that parent meetings which only repeated knowledge present in the information bulletin had limited effect on parenting support. However, greater success was attributed to “theme meetings”; an arrangement similar to coffee table discussions, in which children in junior secondary school, their parents, and school staffs met to discuss and reflect on selected topics, such as mental health, substance abuse, digital medias, etc. One of the participants explained these meetings as follows:

*Then, the pupils and parents are present, and there is such a café dialogue in which they mix. And it is an incredibly nice way to break down some barriers and make contact with other teenagers, and for the teenagers to meet other parents (…) It has been a nice arrangement that has worked incredibly well and in which they have discussed freely. I’m really impressed with how freely they actually talk around the tables* (CTJS 3)

Although common meeting places, both formal and informal, were perceived as important, establishing such common meeting places was not considered sufficient as parenting support. Moreover, there was a requirement that such meeting places contained two-way communication to give participants a real opportunity for creating dialogues and building parent-parent, parent-children, parent-staff, and children-parent-staff relationships.

### Sharing knowledge and language

The last main theme involves understanding the child’s development, growing up in contemporary society, accessing local system knowhow, and sharing a vocabulary that enables adults to address and discuss emotional reactions regarding the children’s psychosocial situation. The subthemes here are *knowledge of the developmental process of children, understanding childhood in contemporary society* and *a common language.*

#### Knowledge of the developmental process of children

Entering parenthood is a first-time experience for all parents. Having knowledge about the normal child developmental phases was viewed as valuable in two regards: In the first place, such knowledge is required to understand children’s needs, reactions, and behaviours during the different phases of their development. For example, having children in junior secondary school was identified as a challenging period in parenting, and participants reported that they saw an increase in the demand for support during this phase:

*When you meet parents that have not had many teenagers before, they express a feeling of complete failure and think that they must give up. Then, when they understand what to expect during this phase and come to know that, probably half of the parents go through the same situation: They become confident (…) We repeat the same message again and again both to the children and the parents; that it is normal to have it this way* (CTJS 1)

During this period, the children are supposed to disengage from their parents and, at the same time, they need parents to guide them into adulthood. Information on what to expect during this phase of children’s development was considered important by participants to normalize the situation.

Second, such knowledge is needed to allow for adults to be prepared to acknowledge developmental challenges and early signs of trouble, so that they can know how to react. This was described in the parent work committee group when discussing successful parent meetings:

External speakers who come to the parent meeting have 15 minutes to talk a little about what research says, what we need to be observant about, and what to look for (P3)

The information from the experts is regarded as knowledge-based advice and is provided universally to parents. Almost as important, participants remarked that experts should endeavour to provide lectures of an appropriate length, namely, short enough to allow for all parents to pay attention and simultaneously have time for reflections at the meeting.

#### Understanding childhood in contemporary society

All participants emphasized the importance of understanding childhood in contemporary society. This is based on the recognition that society changes and influences children’s conditions for growing up. As emphasized by one participant: *“It’s a new world out there; it’s not like before” (SW2).*

Participants explained that they have grown up in another era. Issues such as increase in mental health problems, school dropout, social exclusion, suicide among teenagers, violence in close relations, changing family patterns, new types of drug/substance abuse, and the negative consequences of societal digitization were mentioned as being topics that are currently different from how they were in their era.

The impact of social medias and new forms of digital contact was especially highlighted. Since they grew up during the non-digital period, the participants felt that they were not able to update themselves to utilize the digital applications the children are using: “*There is so much going on online, and we are not able to catch up*” (P3). The new forms of online communication were experienced as particularly demanding:

It is almost impossible to keep oneself updated with the latest app … because new ones come every week, and Yolo is my newest find. It is a type of bully app; and there are hate messages; and you hook up on Snapchat. Obviously, there is always something to keep up with. You must keep up! (SW3)

Thus, understanding the impact of the contemporary society in children was viewed by all stakeholders as important to support the development of robust children who are able to cope with contemporary everyday life.

#### Access to local system knowhow

Participants expressed that parenting support was about receiving—and providing—the needed information to allow for parents to be confident and empowered. They highlighted the importance of parents to have access to available and customized information about the school system, to universal low-threshold parenting support offers, and to knowledge on how to get in touch with available support services when needed. It was particularly essential to know which staff to contact and in what instances these can be consulted when parents have questions, need advice, guidance, or help: “*Who are we supposed to call? Where should we go?* (P1)*”*. Getting sufficient information about where and whom to contact was seen as parenting support.

The need for this practical and contextual parenting knowhow was especially prominent when participants talked about children entering new grades. One of the participants summarized what local system knowhow is about: *“The confidence in knowing how the system works” (CTP3)*

#### A common language

Participants emphasized the importance of children, parents, and professionals to speak a common language. Reportedly, this made it easier to communicate and created a greater sense of confidence for them, as adults, when they had to address and discuss challenging topics regarding the children. Having access to common concepts and to a joint language enabled stakeholders and children to engage in dialogues that addressed sensitive issues. This can be exemplified by an intervention in one of the schools, which introduced the Psychological First Aid Kit to children, parents and staff providing them with a common language to talk about their feelings. One of the concepts was the “red and green thoughts” to understand and address emotional responses:

It was this with red and green thoughts. By then, the kids had also been given that arrangement in class. There was a huge response both in the children and the adults; it allows us to talk of and address emotions more easily (P3)

Mental health, substance abuse prevention, and preventative life management programs were mentioned by participants as interventions that allowed for the establishment of a common language and understanding between stakeholders. Although there were various programs with different concepts and focuses, participants remarked that the value of such interventions came not from the specific programs, but from the fact that all children and adults were given access to a common terminology.

## Discussion

Our findings revealed the experiences of parenting support by stakeholders in a basic school context in Norway. The overall impression of our findings is that parenting support was experienced by our participant groups as a type of support that should be provided to all parents, although the need for such support may vary in degree and time. In the following paragraphs we will discuss our central findings.

### Partnership for the best of the child

The participants in our study experienced parenting support as a reciprocal and a joint activity among parents, peers, and school staff to make parents confident in their parenting role and as equal partners in the home-school collaboration. The focus here was not exclusively on an individual child, but on the fact that children live in a social environment that consists of several actors (i.e., classmates, other parents, school staff, and the child’s family) who interact with them in both informal and formal settings. This finding is in line with and can be explained by Bronfenbrenner’s ecological theory of development (Bronfenbrenner, 1989), in which the child is seen as a part of various interdependent contexts, all of which affect the development of the child (Daly, 2015). Hence, it is imperative that actors in both the home and school context collaborate on issues which affect the child. This collaboration is an important aspect of parenting support according to the participants. Similar findings were reported by Daly and Bray (2015) and is in line with social support and social network theories, which state that social networks provide emotional, instrumental, appraisal, and informative support to those involved, including parents (Belsky, 1984; Berkman & Glass, 2000; Richardson et al., 2007).

In our study, the acceptance of parents as equal partners in the home -school collaboration was considered essential to the experience of parenting support. Geens and Vandenbroeck (2014), stated that when parenting is seen as a shared responsibility between state and citizens, reciprocity and multivocality can come in forefront. In our study, this is related to the stakeholders shared child rearing mission. Thus, parents must be recognized as competent partners that contribute to parenting support activities. This is consistent with Sihvonen (2018), who stated that parents are competent experts in parenting-related issues, implying the need to avoid a hierarchy between parents and professionals. When parents are considered competent experts, horizontal expertise is made available (Sihvonen, 2018). Namely, this allows for peer parents to share their expertise within the school context. Thus, if equality is to be taken as a central element of parenting support, we then need to ask whether the traditional concept of parenting support, in which parents are seen as passive receivers of information, may be misleading. Not recognizing this element of equality may leave a negative impression of parenting support, as Sandbæk (2017) pointed out because parents are then seen as passive and non-competent receivers, rather than resource-orientated equal partners who have the right to self-determination. Having said this, it was expected that the professionals should use their expertise, not by providing general advice on a know-it-all basis, but in line with the findings of Dannesboe et al. (2018). In their study parents viewed the school staff as guides they could turn to both in everyday life and in crisis. As in the research of Dannesboe et al. (2018), we also found that this expertise has to be operationalized and aimed at the child’s and the family’s particular situation. In addition, our study highlights that the value of the expertise depends on the knowledge the professionals have gained about the child and family acquired through daily interactions. Acknowledging that the school and home hold different, yet complementary information about the child, creates opportunities for valuable dialogues on how to provide consistent approaches to support the child (Hodges & Healy, 2018).

Participants also described parenting support as something they did together based on mutual relations that envisioned the best interest of the child. Parents explained how the social networks consisting of other parents, school staff, and pupils were important for them to be confident in their own parenting role. Confidence in the parenting role was in our study dependent on access to a variety of common meeting places, both formal and informal to enable partnerships for the best of the child. This is concurrent with the findings from Daly and Bray (2015), who stated a need for widening the locations in which parenting support is offered. Further, our study underscores that a variety of arenas is not enough. The arenas have to establish and maintain mutual relationships to enable partnerships between peer parents and professionals.

### Capacity to understand and support children in contemporary society

Our study found that what constitutes parenting support differs and is related to the child’s developmental stage and contemporary society. Further, that the need for support varies between the life situation of the child, the parents and the school environment. Participants stated that some phases were more challenging than others and remarked that parenting support should change with the child’s developmental stage and with the influences that society places on the child and family. This is exemplified in our study by the difference between parenting support in primary and secondary school, where the daily contact between home and school declines as the teenagers become more independent and take more responsibility for their own lives. In this regard, we found that the collaboration involved a third party, the pupils themselves, and new measures have to be discovered to create dialogue on creating safe upbringing environments and to take care of each other as fellow human beings in contemporary society. As Sihvonen (2020) has highlighted, our study also revealed the peer community as a valuable resource of support especially for those sharing the same life phase, exemplified here by parenting teenagers. This finding highlights the importance of understanding parenting support as a contextual concept, in that there is no universal understanding of parenting support applicable to all contexts. Our findings are in line with recent studies that draw attention to the contextuality and complexity of the concept of parenting support (Daly, 2015; Frost & Dolan, 2012; Sandbæk, 2017).

Receiving and providing relevant information was considered by our participants as crucial for establishing and maintaining a successful and effective collaboration between home and school. Informational support has been highlighted in prior studies as an element in parenting support (Belsky, 1984; Berkman & Glass, 2000; Richardson et al., 2007). Participants in this study, highlighted the mutual need for sharing information to enable each other in joint efforts to support the children. Information was understood in a broad sense including e.g., knowledge of normal behaviour, early signs of trouble, system knowhow, how to manage challenges related to the specific child and its particular life situation as well as personally gained knowledge from interacting with the child at home and in school. This is consistent with findings from Daly (2015), who stated that sharing information about good parental practice was one of the three elements of parenting support. For our participants information was not only about information from experts about best parenting practices, but also the mutual exchange of information between parents, peers and the school from their daily interactions was vital.

Finally, the use of a common language by children, parents and professional was highlighted as important to enhance dialogues addressing emotional responses and sensitive topics. Having a common language and shared terminology may function in two ways; as an awareness and understanding of the children’s emotional responses and by this address and prevent emotional problems at an early stage.

### Parenting support as reciprocal, contextual and multidimensional

Based on the results of this study the concept of parenting support was experienced as reciprocal, contextual, and multidimensional.  “Reciprocal” meaning that all stakeholders in parenting support should work towards the common goal of serving the best interest of the child, which presupposes a collaboration between the adults surrounding the child. ’Contextual’ means that the developmental phase of the child, the home-school collaboration, the impact of the contemporary society on the child, and the requirements that these issues bring to the parental role should be regarded during parenting support. Finally, ’multidimensional’ means that parenting support consists of several elements, which correspond to the main themes in this study: A community for the best of the child, uniting through relations, and sharing knowledge and language.

This study highlights the need to view parenting support as an integral part of the collaboration between school, home, and the parenting community towards achieving the child’s best interest. This implies the need to shift the targeting of parenting support from support to families at risk and non-competent parents in need of an intervention—which place parents as passive recipients—to all parents, and the need to acknowledge parents as competent, resourceful, and active partners. Understanding parenting support as a continuous and integrated part of the home-school collaboration facilitates interventions that focus on empowerment and resource provision based on the specific environment in which this support will be applied. Such a perspective has implications for policy, practice and further research.

## Implications

On the policy level, this study calls for acknowledging the individuality of children, their parents and life contexts and not presume that standardized programs based on parental needs predefined by experts are the best option.

To empower parents as partners, the school management level should endeavour to provide the staff with time and resources to engage in parenting support activities daily to promote the school-home-peer community collaboration. We believe this approach may allow for more effective parenting support interventions. According to our findings, the use of the concept of parenting support, when literally interpreted, may be misleading to the experiences of parenting support in a school context. Our study participants situated universal parenting support in the context of mutual collaboration and relations for the best of the children. This is incompatible with the literal understanding of parenting support as something given to parents by experts.

Thus, adverse situations and new developmental stages call for specific support for parents and targeted interventions both at an individual and group level to enable confident parents. Maybe, the concept of parenting support should be reserved for these activities? If so, taking the school- home partnership as the position of departure may enable parents to be actively engaged and empowered as the most important persons in the children’s lives and also support the school in their common and highly important child rearing mission.

## Strengths and limitations

This study contributes to the ongoing discourse on parental support by presenting empirically based knowledge from those involved at the executive level in the schools on their experiences of parenting support activities and how this forms an understanding of what constitutes parenting support. Of particular value is the insight in the understanding of parenting support as orientated by the best of the children and viewed as a collaboration between all the adults who relate to the child at school and home. These findings indicate that the concept of parenting support from a street-level perspective should be an integral part of the everyday school- home-parenting community collaboration rather than isolated activates targeting parents with expert knowledge in adverse situations.

This study has a limited number of participants. According Malterud et al. (2016), the concept of information power requires that the more information the sample holds relevant for the actual study, the lower the number of participants needed. The data from our study is rich in information and had examples from the participants’ own experiences, both as by being parents themselves and in their diverse roles.

As parenting support measures target parents in general, the lack of perspective from lay parents without roles in the formalized school-home collaboration committees is a limitation in our study. The lay parents’ perspectives may likely differ from our participants’ views; hence, imperative that lay parents’ views are included in future studies to get a full understanding of the concept.

Methodologically, the data was generated in homogenous focus groups across schools, which may have led the participants to a greater agreement than would have been the case in individual interviews. Yet, the idea behind the focus group method is that group processes can help people to explore and clarify their views in ways that would be less easily accessible in a one to one interview (Kitzinger, 1995). As our study found no clear differences between the groups, we believe that the interaction with others might have enabled them to vocalize their views on what constitutes parenting support. Thus, since there were few critical statements in the groups and more harmonizing answers, we suggest that the more critical aspects of parentings support measures in school contexts should be specifically addressed in future studies.
